# Radiation-induced DNA double-strand breaks in peripheral leukocytes and therapeutic response of heel spur patients treated by orthovoltage X-rays or a linear accelerator

**DOI:** 10.1007/s00066-020-01662-4

**Published:** 2020-07-10

**Authors:** Sebastian Zahnreich, Hans-Peter Rösler, Carina Schwanbeck, Heiko Karle, Heinz Schmidberger

**Affiliations:** Department of Radiation Oncology and Radiotherapy, University Medical Center, Mainz, Germany

**Keywords:** Radiotherapy, Benign disease, Heel spur, Biodosimetry, γH2AX

## Abstract

**Purpose:**

Biodosimetric assessment and comparison of radiation-induced deoxyribonucleic acid (DNA) double-strand breaks (DSBs) by γH2AX immunostaining in peripheral leukocytes of patients with painful heel spur after radiation therapy (RT) with orthovoltage X‑rays or a 6-MV linear accelerator (linac). The treatment response for each RT technique was monitored as a secondary endpoint.

**Patients and methods:**

22 patients were treated either with 140-kV orthovoltage X‑rays (*n* = 11) or a 6-MV linac (*n* = 11) with two weekly fractions of 0.5 Gy for 3 weeks. In both scenarios, the dose was prescribed to the International Commission on Radiation Units and Measurements (ICRU) dose reference point. Blood samples were obtained before and 30 min after the first RT session. γH2AX foci were quantified by immunofluorescence microscopy to assess the yield of DSBs at the basal level and after radiation exposure ex vivo or in vivo. The treatment response was assessed before and 3 months after RT using a five-level functional calcaneodynia score.

**Results:**

RT for painful heel spurs induced a very mild but significant increase of γH2AX foci in patients’ leukocytes. No difference between the RT techniques was observed. High and comparable therapeutic responses were documented for both treatment modalities. This trial was terminated preliminarily after an interim analysis (22 patients randomized).

**Conclusion:**

Low-dose RT for painful heel spurs with orthovoltage X‑rays or a 6-MV linac is an effective treatment option associated with a very low and comparable radiation burden to the patient, as confirmed by biodosimetric measurements.

## Introduction

The successful treatment of benign inflammatory and degenerative conditions like calcaneodynia (painful heel spur) and painful shoulder or elbow syndrome with low-dose radiation therapy (RT) has a longstanding history in Germany and accounts for more than a third of all RT patients treated annually [1]. Local administration of photon doses below 1 Gy has been shown to attenuate inflammatory responses as the causative factor for these painful diseases [2]. Clinical studies documented comparable positive results for treatment schedules with single fractions of 0.5 Gy or 1 Gy applied twice a week for 3 or 6 weeks [3–5]. Accordingly, the iso-effective single dose of 0.5 Gy over a course of 3 weeks is used for radiation protection purposes to reduce the radiation burden to the patient and the risk of potential radiation-induced (RI) stochastic late effects such as radiation carcinogenesis [6]. The risk of adverse late effects induced by low-dose RT for benign diseases is generally considered to be negligible and the benefit for the patient outweighs [7–9]. Anthropomorphic phantom-based studies by Jansen et al. [10] using an effective dose concept for a cumulative dose of 6 Gy administered in six fractions of 1 Gy predict rough estimations for the risk of RI fatal tumors of 20–40 per 1000 patients for heterotopic ossification, 1.5 per 1000 patients for gonarthrosis, 0.5 per 1000 patients for heel spurs, and 1 per 1000 female patients for hidradenitis suppurativa. Other predominant factors that influence the risk of radiation carcinogenesis besides the target dose are the field size, photon energies, the exposed anatomic region/organs, sex, and age at exposure. In general, such risk estimates are fraught with large uncertainties in the order of a factor of 2 and are expected to be much less for low-dose RT of benign diseases, since this treatment is predominantly performed in elderly patients with a strongly and up to nine-fold reduced excess lifetime risk for RI solid tumors when compared to young adults [10–12].

Calcaneodynia is observed in 10–15% of the population and is associated with severe heel pain causing restrictions and reduced quality in everyday life [13, 14]. In Germany, approximately 10,000 heel spur patients per annum are treated by low-dose RT in about 340 active facilities offering RT for benign diseases [15]. Treatments are usually conducted with a medical linear accelerator (linac) and megavoltage (MV) photons or orthovoltage devices delivering X‑rays in the range of 100–400 keV, with a prevalence for linacs [15]. Compared to an orthovoltage device, RT with a linac achieves a more homogeneous dose distribution in the target volume, which has been discussed to have improved therapeutic effectiveness [16]. However, the penetration depth of MV photons from a linac exceeds the cross-section of the patient and causes reflections in the RT bunker that contribute to an inevitable dose burden to the patient’s normal tissue outside the target volume. So-called out-of-field doses are also generated by scattering and leakage from the linac treatment head, collimation devices, and, to a lesser extent, by scattering from within the patient’s body [17]. Such low-dose exposures far from the primary beam have been associated with various radiation-related late adverse effects [18, 19]. Orthovoltage RT with low-energy photons has an inferior dose distribution but allows for protection of the patient’s healthy tissue by lead shielding which cannot be applied for high-energy photon units due to the generation of scatter radiation within the shielding equipment. However, for linac RT, treatment planning including field collimation can be conducted to protect the patients’ healthy tissue outside the target volume.

Besides conventional physical measurements to determine the patient’s radiation exposure, the detection of biological indicators of RI deoxyribonucleic acid (DNA) damage in peripheral leukocytes has been frequently applied for biodosimetric purposes in patients undergoing various radiologic procedures or RT [20–29]. By far the most sensitive and rapid measure is the immediate quantification of RI DNA double-strand breaks (DSB) using the phosphorylated histone variant H2AX (γH2AX) or tumor protein 53 binding protein 1 (53BP1), representing well-established surrogate markers of DSBs [30]. Immunostaining and fluorescence microscopic quantification of so-called γH2AX or 53BP1 foci at the level of single cells is proportional to the number of RI DSBs and therefore increases linearly with radiation dose after ex vivo and in vivo exposure with a detection threshold of a few mGy [22, 26, 27].

However, it is hitherto unknown whether the largely random application of linac or orthovoltage RT for the treatment of painful heel spur is associated with a varying unwanted but inevitable radiation exposure of the patients’ normal healthy tissue to out-of-field doses and differing therapeutic effectiveness. Therefore, we conducted this prospective randomized trial as a biodosimetric approach to assess the radiation burden of heel spur patients treated either with orthovoltage X‑rays or a linac by quantitation of the DSB marker γH2AX in peripheral leukocytes. RT was given twice per week with a single dose of 0.5 Gy and six total fractions. Venous blood samples were drawn immediately before and 30 min after the first fraction of RT and were processed for fluorescence microscopic scoring of γH2AX foci in leukocytes. As a secondary endpoint the analgesic effectiveness was monitored based on a five-level function calcaneodynia score (CS) before and 3 months after RT.

## Patients and methods

### Patients and radiation therapy

**Fig. 1:**
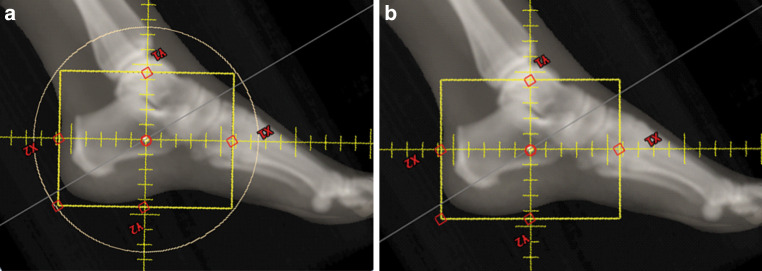
Exemplary representation of radiation therapy (RT) for plantar fasciitis treated either by **a** orthovoltage X‑rays performed with a round mechanical applicator with a diameter of 15 cm or **b** linac RT. The treatment field was shaped for orthovoltage therapy using lead rubber shielding and for linac RT using the aperture only with no collimation. Simulated radiographs are shown since no computed tomography-based treatment planning was performed

Patients suffering from painful heel spur were enrolled based on the following inclusion criteria: radiologic evidence of spur formation, anamnesis of a painful heel and functional impairment, painful symptoms for a least 3 months, age ≥40 years, overall condition allowing for repeated venous blood sampling, and a signed an informed consent form approved by the local ethics committee. The following exclusion criteria were applied: known gene defects associated with compromised DNA repair (e.g., ataxia–telangiectasia, Werner syndrome, or Bloom syndrome), age <40 years, previous RT or trauma in the treated anatomical region, any exposure to ionizing radiation less than 5 days before the start of RT, any additional inflammatory or rheumatic disease, pregnancy, breastfeeding, intellectual disability or psychiatric disorder, legal care in health matters, and lack of a signed informed consent form approved by the local ethics committee. The characteristics of the participants are summarized in Table 1. Based on these criteria, 22 patients (36%) of a total number of 61 entered the study between August 2016 and August 2019, and were randomized into two groups: 11 patients (50%) were treated with orthovoltage X‑rays (140 kV, D3150 X‑Ray Therapy System, Gulmay Ltd., Byfleet, UK) and 11 patients (50%) were treated with a linac (6 MV, Clinac DHX, Unique^TM^ or Truebeam® Varian Medical Systems, Palo Alto, CA, USA) at the Department of Radiation Oncology and Radiation Therapy at the University Medical Centre Mainz, Germany. All patients received two weekly fractions of 0.5 Gy applied as two lateral opposing fields up to a total dose of 3 Gy. The calcaneus and the plantar aponeurosis were included in the target volume. The average field size for both RT techniques was 8 × 10 cm^2^. For orthovoltage RT the field size was defined by a mechanical applicator with a diameter of 15 cm to collimate the beam and set the source-to-skin distance (SSD) and was further adjusted using lead rubber shielding. For linac RT the treatment field was defined by the aperture with no collimation, since no computed tomography-based treatment planning was performed. Representative treatment planning images for both RT techniques are shown in Fig. 1. The dose rate for orthovoltage RT was 3.64 Gy per minute and approximately 3 Gy per minute for linac RT, resulting in comparable average beam-on times of 0.17 and 0.19 min, respectively. The average SSD for orthovoltage and linac RT was 25 cm and 97 cm, respectively. The study was approved by the Ethics Committee of the Medical Association of Rhineland-Palatinate, number 837.216.15 (9984) on 07/30/2015, and by the expert committee of the DEGRO (German Society for Radiation Oncology). Patients were assigned to one of the RT techniques by block randomization with an equal probability for both arms. According to the results of our previous biodosimetric studies based on γH2AX foci quantification in peripheral leukocytes after fractionated RT of prostate and breast cancer patients [26, 27], 60 patients are required to detect a difference of 25% with a power of 80% and an error probability of 5%. To detect a difference of 10% in the CS scores with a power of 80% and an error probability of 5%, we estimated a total number of 120 participants. This trial was terminated preliminarily after an interim analysis (22 patients randomized).

**Table 1:** Patient characteristics and previous treatments

Criteria	All patients *n* (%)	Orthovoltage *n* (%)	Linac *n* (%)
*Patients*	22	11 (50%)	11 (50%)
*Sex*			
Females	14 (64%)	6 (55%)	8 (73%)
Males	8 (36%)	5 (45%)	3 (27%)
*Age (years)*			
Median (range)	54 (40–77)	57 (40-77)	53 (42–69)
*Site*			
Left	11 (50%)	7 (64%)	4 (36%)
Right	11 (50%)	4 (36%)	7 (64%)
*Duration of symptoms (months)*			
Median (range)	9 (2–36)	8 (2–36)	9 (3–36)
≤6 months	7 (35%)	4 (40%)	3 (30%)
>6 months	13 (65%)	6 (60%)	7 (70%)
*Previous treatments*			
Insoles	1 (5%)	0 (0%)	1 (9%)
NSAID	5 (23%)	3 (27%)	0 (0%)
ESWT/ultrasound	7 (32%)	6 (55%)	1 (9%)
Corticoid infiltration	3 (14%)	10 (91%)	2 (18%)
Heel pad	17 (77%)	10 (91%)	7 (64%)
TENS	1 (5%)	1 (9%)	0 (0%)
Ice bag	3 (14%)	1 (9%)	2 (18%)

*Linac* linear accelerator,* ESWT* extracorporeal shock wave therapy, *NSAID* nonsteroidal anti-inflammatory drugs, *TENS* transcutaneous electrical nerve stimulation

### Treatment response

The treatment response was documented based on standardized questionnaires using a five-level functional CS score according to [3, 31] before and 3 months after the first course of RT. Data were available for 20 patients pre-RT and 19 patients post RT.

### Blood sampling and ex vivo irradiation

Venous blood collection, irradiation of whole blood, and isolation of leukocytes were performed as described previously [26, 27]. Venous blood was drawn immediately before and 30 min after the first fraction of RT. Blood samples taken before RT were sham irradiated or exposed to a test dose of 0.5 Gy X‑rays and analyzed 30 min after irradiation to assess the individual yield of basal or RI DSBs by γH2AX foci quantitation, respectively. Ex vivo irradiation of whole blood was performed with a D3150 X‑Ray Therapy System (Gulmay Ltd., Byfleet, UK) at 140 kV and a dose rate of 3.6 Gy per min at room temperature. Sham-irradiated cells were kept under the same conditions in the radiation device control room.

### γH2AX foci quantification

**Fig. 2:**
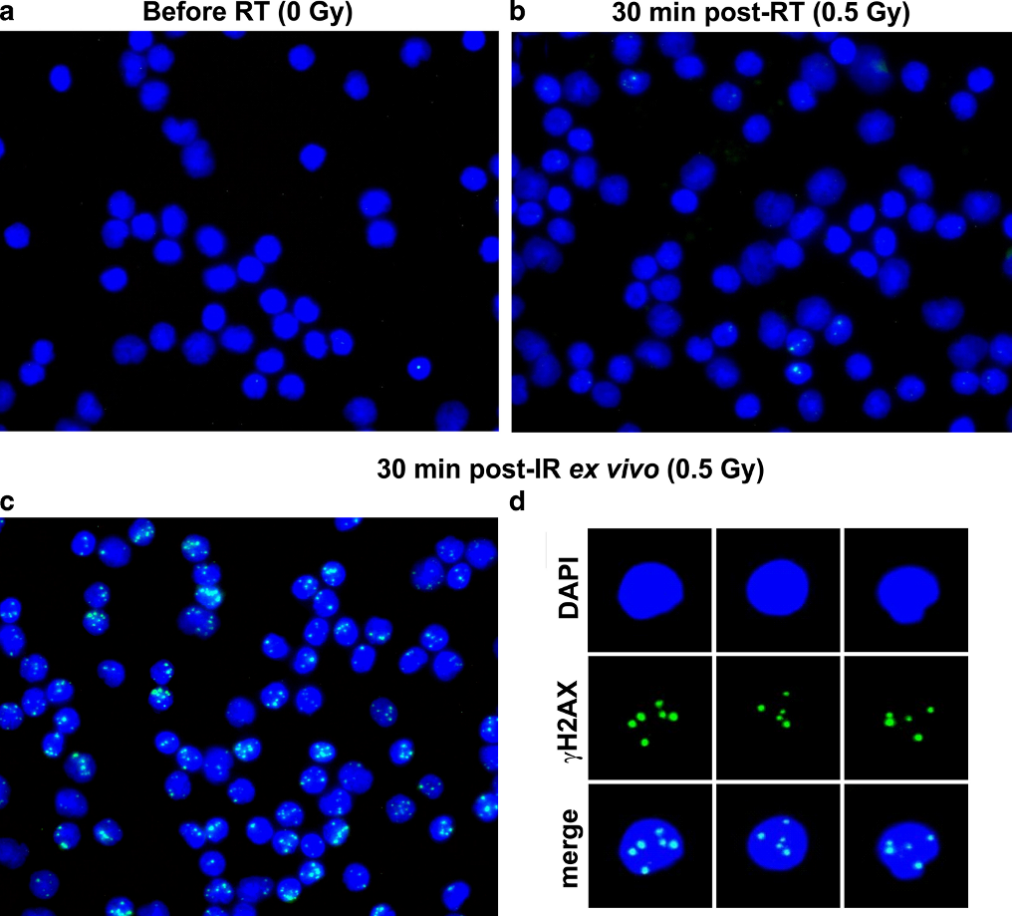
Immunofluorescence staining for γH2AX (*green*) in DAPI-stained (*blue*) nuclei of peripheral leukocytes 30 min after **a** sham irradiation, **b** the first fraction of radiation therapy (RT) or **c**, **d** homogeneous ionizing radiation (IR) exposure ex vivo. **d** Nuclei extracted from **c** as used for manual counting of γH2AX foci at the level of single cells

Fixation of leukocytes, γH2AX immunostaining, fluorescence microscopy, image capturing, and scoring of foci was performed as described previously [26, 27]. After irradiation ex vivo or in vivo, at least 100 or 1000 cells were analyzed manually for each datapoint, respectively. Representative immunofluorescent images for γH2AX foci quantitation are shown in Fig. 2.

### Equivalent whole-body dose estimation

To approximate an equivalent whole-body dose (EWBD) for each patient according to [32], calculations were performed based on the formula:

$$\text{Integral dose}=(1.44\times D_{0}\times d_{1/2}\times A\times [1-\exp(-0.6938d/d_{1/2})]\times (1+2.88d_{1/2}/f))/1000$$

where *D*_*0*_ represents the administered dose (Gy), *A* the field area (cm^2^), *d* the patient diameter (cm), *d*_*1/2*_ the half-value thickness (140 kV = 5 cm and 6 MV = 15 cm), and *f* the SSD (cm). The formula was applied to each treatment field to obtain an integrated dose that was divided by the patient’s weight to obtain the EWBD.

### Data and statistical analysis

For quantification of γH2AX foci in leukocytes a single sample was available and analyzed per datapoint for each patient. Due to the limited amount of blood, no biological or technical replicates could be performed. All data were scored by one observer. To obtain the numbers of RI foci only, the individual basal yield of γH2AX foci was subtracted from the yield after ex vivo or in vivo irradiation. Summarized patient data are provided as the mean and standard deviation unless stated otherwise. Data handling, plotting, and statistics were performed using SigmaPlot^TM^11® (Systatt Software Inc., San Jose, CA, USA). The relationship between two variables was analyzed using the Pearson correlation. For comparison of two groups the Student’s *t*-test or the Mann–Whitney rank sum test was used. The comparison between two categorical variables was performed using the Fishers’ exact test. All levels of significance were set at *p* < 0.05.

## Results

### γH2AX foci before radiation therapy and after irradiation ex vivo

**Fig. 3:**
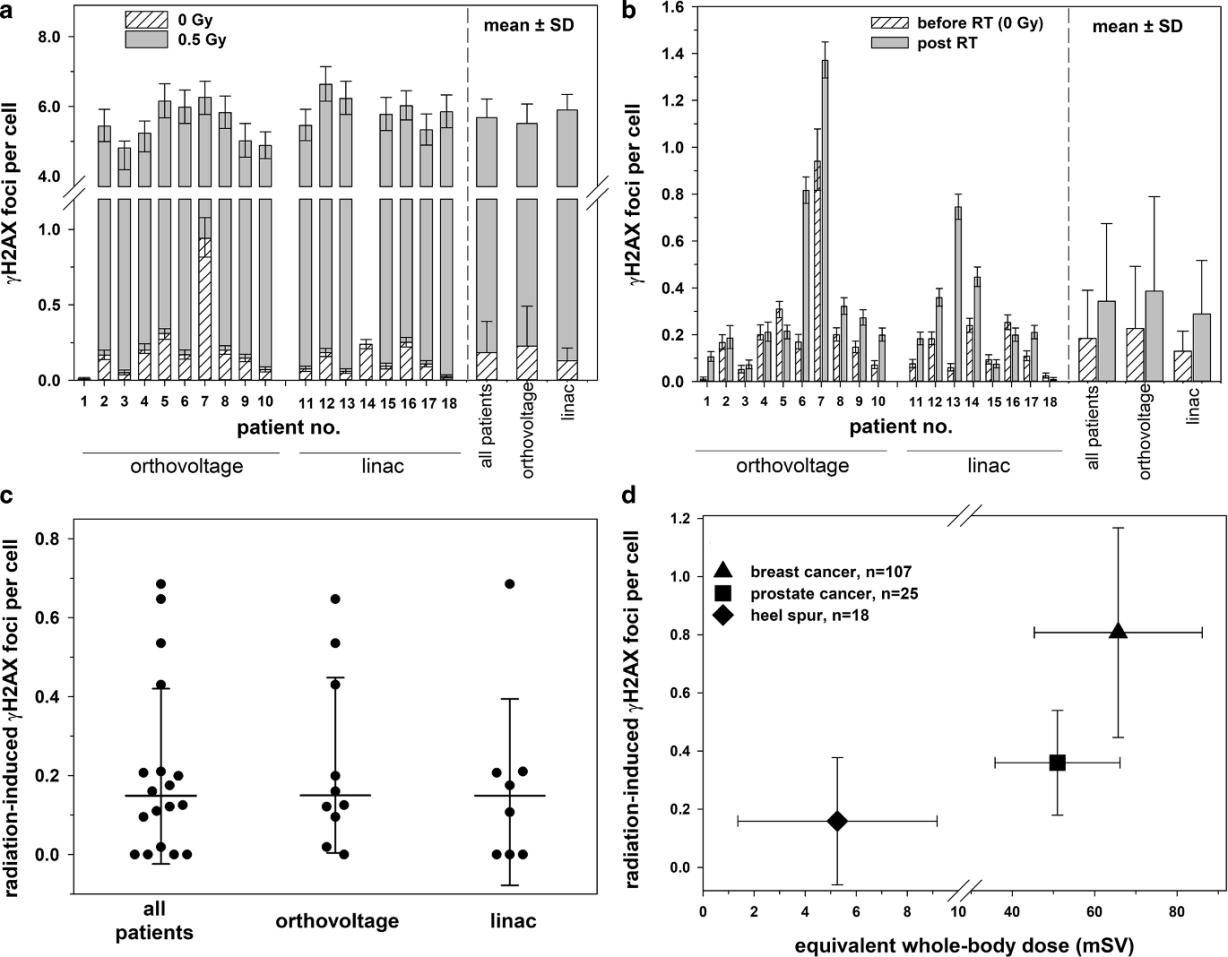
Scoring of γH2AX foci in patients’ leukocytes. Average numbers of γH2AX foci per leukocyte in sham-irradiated cells before radiotherapy (RT) and **a** 30 min after ex vivo exposure to 0.5-Gy X‑rays or **b** 30 min after RT for each patient as well as the respective mean ± standard deviation (SD) of all patients (*n* = 18) and patients treated by orthovoltage (*n* = 10) or linac (*n* = 8) RT. Ex vivo irradiated samples of donors 1 and 14 were not available. Error bars for individual patients represent the 95% confidence interval of the Poisson mean. **c** Average numbers of radiation-induced γH2AX foci per leukocyte 30 min after the first fraction of RT in all patients and patients treated by orthovoltage RT or linac RT only. *Dots* show the individual values of each patient. *Solid lines* represent the mean and *error bars* the SD. **d** Comparison of the average yield of radiation-induced γH2AX foci per leukocyte in all heel spur patients of the present study and in tumor patients as obtained in our previous studies [26, 27] in dependence of the administered equivalent whole-body dose. *Error bars* represent the SD

To examine the individual basal level of DSBs and the ex vivo radiation response in patients’ leukocytes, blood samples obtained before RT were sham irradiated or exposed to 0.5 Gy X‑rays and analyzed 30 min post radiation. The results are shown in Fig. 3a and detailed information on γH2AX foci quantitation are provided in Table 2. The average rate of basal γH2AX foci in leukocytes scored in 18 patients was 0.184 ± 0.206 per cell. The endogenous level of γH2AX foci in leukocytes before RT did not correlate with the patients’ age (r = 0.346, *p* = 0.160), gender (r = −0.159, *p* = 0.528), or CS sum score prior to RT (r = −0.063, *p* = 0.817). 30 min after exposure to 0.5-Gy X‑rays ex vivo we observed a comparable induction of γH2AX foci per leukocyte for all patients. On average, 5.76 ± 0.68 γH2AX foci per cell were scored in 16 analyzable patient samples corresponding to an excess of 5.57 ± 0.56 RI foci per cell. Thus, despite large variations in the individual background number of γH2AX foci in patients’ leukocytes, a more uniform and comparable response after homogeneous ex vivo exposure to ionizing radiation was observed. There was no significant difference in the average yield of basal (*p* = 0.625) or ex vivo RI (*p* = 0.223) γH2AX foci per leukocyte between the two RT groups.

**Table 2:** γH2AX foci per cell before RT, 30 min after ex vivo exposure to 0.5 Gy or 30 min after the first fraction of RT

RT	EWBD (mSV)	γH2AX foci per cell		
		Before RT 0 Gy	Ex vivo 0.5 Gy	Post RT
Orthovoltage	2.52	0.011	n.a.	0.106
	2.36	0.167	5.44	0.186
	2.79	0.051	4.81	0.072
	3.49	0.200	5.23	0.211
	2.44	0.310	6.15	0.215
	2.55	0.169	5.98	0.816
	3.24	0.940	7.32	1.371
	3.15	0.200	5.82	0.321
	2.34	0.147	5.02	0.272
	2.39	0.071	4.88	0.199
Mean ± SD	2.73 ± 0.42	0.227 ± 0.265	5.63 ± 0.80	0.377 ± 0.406
Linac	9.99	0.076	5.46	0.183
	13.48	0.183	6.81	0.358
	9.97	0.060	6.23	0.745
	14.67	0.239	n.a.	0.446
	10.96	0.093	5.77	0.074
	13.30	0.252	6.02	0.199
	9.50	0.109	5.33	0.210
	12.63	0.025	5.84	0.010
Mean ± SD	11.81 ± 1.95	0.130 ± 0.085	5.92 ± 0.499	0.278 ± 0.235
All patients Mean ± SD	5.26 ± 3.90	0.184 ± 0.206	5.76 ± 0.680	0.333 ± 0.335

*RT* radiation therapy*, EWBD* equivalent whole-body dose, *SD* standard deviation, *Linac* linear accelerator

### γH2AX foci after radiation therapy

After the first session of RT the frequency of γH2AX foci per leukocyte exceeded the background rate in 78% (14/18) of all patients. Increments were detected in 90% (9/10) of patients after orthovoltage RT and 63% (5/8) of patients after linac RT, with no significant difference between the treatment arms (*p* = 0.725). Fig. 3b, c show the level of γH2AX foci before and after the different in vivo exposure scenarios and detailed information is provided in Table 2. After RT the rate of γH2AX foci per leukocyte increased significantly from the average basal number of 0.184 ± 0.206 to 0.333 ± 0.335 (*p* = 0.045) for all patients, corresponding to an excess of 0.149 ± 0.222 RI foci per cell. No significant increment of γH2AX foci after RT was observed within the orthovoltage (*p* = 0.089) or linac (*p* = 0.091) arm only. In patients treated with orthovoltage X‑rays or a linac, the average rate of RI γH2AX foci post RT reached a comparable level of 0.150 ± 0.222 or 0.149 ± 0.236, respectively (*p* = 0.51). However, the average EWBD of linac RT (11.81 ± 1.95 mSV) was significantly higher than for orthovoltage RT (2.73 ± 0.42 mSV; *p* < 0.0001, Table 2). Accordingly, we observed no correlation between the calculated EWBD and the yield of RI γH2AX foci per cell post RT (r = −0.074, *p* = 0.769).

To evaluate the patient’s radiation burden during RT of heel spurs, Fig. 3d shows the mean frequencies of RI γH2AX foci 30 min after RT from this work related to data from our previous biodosimetric studies 30 min after RT of breast or prostate cancer patients with an average single dose of 2 Gy [26, 27]. Compared to the mean number of RI γH2AX foci in leukocytes after heel spur RT with a single dose of 0.5 Gy (0.159 ± 0.227, *n* = 18), the rate of γH2AX foci was 2.4- or 5.5-fold higher after the first fraction of prostate (0.360 ± 0.0180, *n* = 25) or breast (0.807 ± 0.360, *n* = 107) cancer RT, respectively.

### Treatment response

**Fig. 4:**
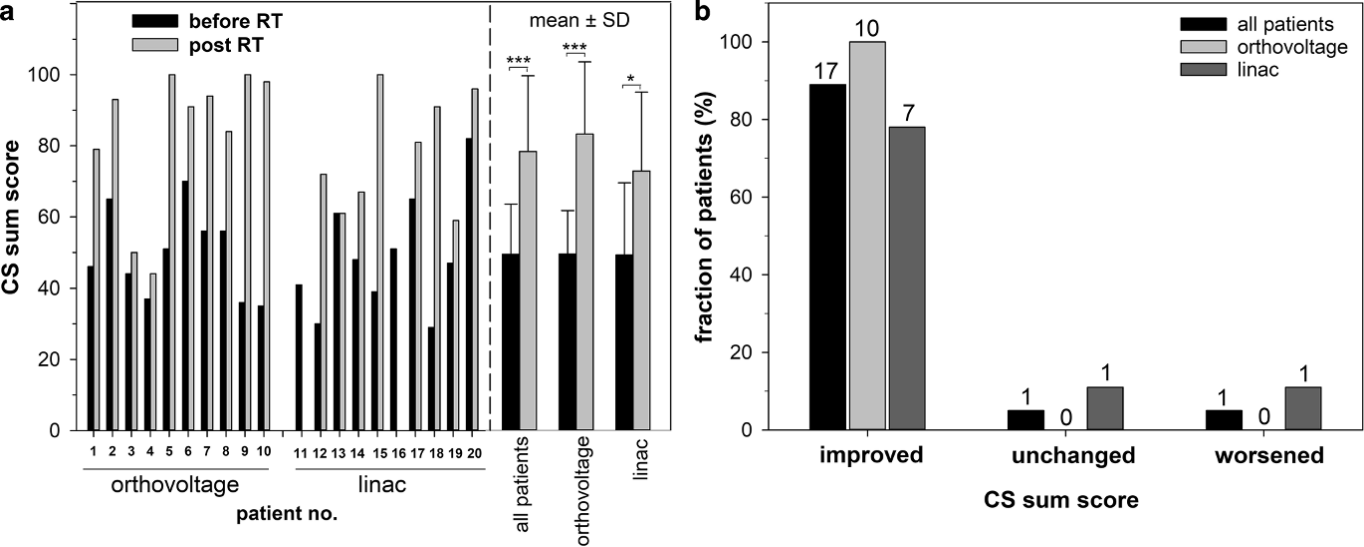
Variation of the summed calcaneodynia score (CS) before and at 3 months after radiotherapy (RT) for **a** each patient and compiled for all patients or orthovoltage RT or linac RT only. **b** Summarized data on changes in the sum score 3 months after RT. Numbers of patients are indicated. The CS score of patient no. 16 at a 3-month follow-up was not available. Statistical comparisons between two groups were performed by the student’s t‑test. *SD* standard deviation, *p < 0.05, **p < 0.01, ***p < 0.001

For each patient the CS single score criteria and the sum score were assessed before and 3 months after RT and are shown in Fig. 4a. The average CS sum score for all patients before RT was 49.5 ± 14.1 and was comparable for patients treated with an orthovoltage device (49.6 ± 12.2) or a linac (49.3 ± 16.4; *p* = 0.963). At 3 months follow-up we observed a significant 1.6-fold increase of the CS sum score up to 78.4 ± 14.1 for all patients (*p* < 0.001) and to 83.3 ± 20.3 (*p* = 0.017) or 72.9 ± 22.2 (*p* = 0.017) in the orthovoltage or linac arms, respectively. There was no significant difference in the sum score post RT between patients treated by orthovoltage or linac RT (*p* = 0.301). 89% (17/19) of patients in both arms had an improved sum score and unchanged or worsened conditions were reported by only 5.5% (1/19) of the patients each (Fig. 4b). After orthovoltage RT all patients had an elevated sum score, whereas after linac RT the sum score increased in 78% (7/9) of patients only and was unchanged or worsened in 11% (1/9) of the patients each. Statistical comparison of the proportions of patients with an improved sum score showed no significant difference between the two RT groups (*p* = 0.211).

**Fig. 5:**
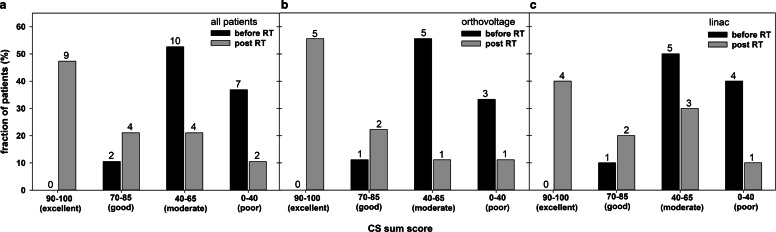
Performance status according to categories of the summarized calcaneodynia score (CS) before and 3 months after radiotherapy (RT) for **a** all patients or patients treated by **b** orthovoltage RT or **c** linac RT. Numbers of patients are indicated

Fig. 5 shows the distribution of patients classified in the performance categories excellent (CS sum score 90–100), good (CS sum score 70–85), moderate (CS sum score 45–65), or poor (CS sum score 0–40). Before RT, none of the patients were classified as excellent and only 10% (2/19) as good (Fig. 5a). The majority of patients, 53% (10/19) and 37% (7/19), were in the categories moderate and poor, respectively. Before RT the patients were distributed similarly among the four categories between both RT techniques (Fig. 5b, c). At 3 months follow-up, 47% (9/19) of all patients were ranked as excellent, 21% (4/19) as good or moderate each, and the remaining 11% (2/19) as poor (Fig. 5a). After orthovoltage RT, 56% (5/9) of the patients were in the category excellent, 22% (2/9) in good, and 11% (1/9) in moderate or poor each (Fig. 5b). After linac RT, 40% (7/10) of patients were in the category excellent, 20% (2/10) in good, 30% (3/10) in moderate, and 10% (1/10) in poor (Fig. 5c). No correlation was found between an improved sum score and patient’s age (r = 0.439, *p* = 0.0601), duration of painful symptoms (r = 0.228, *p* = 0.347), or gender (r = 0.259, *p* = 0.285). Information on patient characteristics is provided in Table 1.

## Discussion

In the present study we measured RI DSBs by γH2AX foci quantification in peripheral leukocytes of painful heel spur patients treated with a 140 kV orthovoltage device or a 6-MV linac to assess the patients’ radiation burden for radiation protection purposes. Immediately after the first fraction of RT we detected an overall slight but significant increase of γH2AX foci with no difference between orthovoltage and linac RT. The application of either RT technique led to significant and comparable pain relief at 3 months follow-up. Based on this outcome as well as low participation and inclusion rates, the trial was terminated preliminarily after an interim analysis (22 patients randomized).

DSBs are potently induced by ionizing radiation and represent the most deleterious DNA lesion causing cell death, chromosomal rearrangements, and malignant transformation [33]. The by far most prominent biomarker of RI DSBs is the phosphorylated histone variant γH2AX, which has been applied in numerous studies to evaluate the in vivo radiation exposure of patients after low-dose radiologic examinations like computed tomography (CT) (e.g., [20, 22, 23, 34]) and mammography [21, 25], or after high-dose RT of tumor patients [24, 26–29]. The present study is the first, at least to our knowledge, to apply this method with a biodosimetric intention in patients treated by low-dose RT for benign inflammatory and degenerative diseases with pain relief as the second clinical endpoint. RT of this medical condition is very well received and frequently used in German-speaking regions but is barely applied in other, particularly Anglo-American, countries [1]. Such geographical differences are due to fear of RI late adverse effects, which, however, are estimated to be very low or negligible for this type of local low-dose RT [7, 8]. Various studies on the RT of heel spurs showed equal effectiveness for single doses of 1 Gy or 0.5 Gy administered twice a week for 3 or 6 weeks [3–5, 16]. Accordingly, the lower dose of 0.5 Gy represents the standard option to decrease the potential risk for radiation-related late adverse effects [6]. Depending on the institutional equipment, benign diseases are treated either with a linac and MV photons or with an orthovoltage device operating in the low-energy kV range. This instrumentation-specific difference might be associated with varying therapeutic effectiveness [16] but also with divergent exposures to undesirable out-of-field doses [17]. Also, from a health economic perspective, orthovoltage RT is associated with lower costs compared to linac RT. Based on this rationale, we investigated both endpoints in this prospective randomized trial after low-dose RT of calcaneodynia patients with a linac or orthovoltage unit for a treatment schedule of 0.5 Gy given twice a week over a course of 3 weeks. According to our scoring criteria adapted from Rowe et al. [31] and Heyd et al. [3], we observed high response rates and pain reduction in up to 89% of patients at 3 months follow-up in line with improvement rates of previous studies ranging between 65–100% [16]. About 50% of patients reported on an excellent and pain-free performance status after the first RT series. We did not observe any difference in the therapeutic response between the two RT techniques, but the small number of participants does not allow meaningful statistical comparisons. Previously, Muecke et al. [16] performed a retrospective study on the long-term treatment success of low-dose RT for painful heel spurs in 502 patients treated either with 6–10-MV photons twice per week or with 175 kV X‑rays three times per week at four different facilities in Germany. Patients received 10 fractions of 0.5 Gy or 5–6 fractions of 1 Gy for 6–10-MV photons or six fractions of 1 Gy for orthovoltage X‑rays. In their study, multivariate analysis revealed a significantly worse prognosis for orthovoltage RT than for MV photons, with no impact of radiation dose. This finding has been related to a more homogeneous and favorable dose distribution achieved with MV units. No other study has yet confirmed this observation. Although a better distribution of dose is achieved in the target volume for linac RT, it may increase the radiation burden of the patient through higher peripheral doses outside the primary beam caused by radiation scattering, leakage, and reflections [17]. Besides physical dosimetry, biodosimetric attempts have been made to compare the inherent radiation exposure of different RT techniques [27–29] or CT protocols [35] based on the quantification of RI γH2AX foci in peripheral leukocytes. Thresholds for this highly sensitive assay to monitor the induction of RI DSBs in vitro and in vivo have been set at 1 mGy and 3 mGy, respectively [22, 36]. So far, only few comparable studies on foci quantification of DSB repair proteins in systemic lymphocytes after a planned medical IR exposure in vivo are available for the low EWBDs of the present work, which were able to demonstrate dose-dependent increments or even differences between radiation techniques. Kuefner et al. [35] reported on significantly reduced levels ofγH2AX foci in peripheral leukocytes 30 min after multidetector coronary CT angiography performed with a dose-reducing sequential protocol compared to a conventional helical protocol in line with physical dose estimates. For an approximated median effective dose ranging from 2.1 to 23.8 mSv, the authors described a linear dose response for the induction of excess γH2AX foci in vivo from 0.04 to 0.71 foci per leukocyte with a median of 0.33 in line with similar studies [22]. In another study these authors investigated the impact of digital mammography executed with doses even lower than for CT examinations [25]. Again, a very slight but significant increment of γH2AX foci was found in systemic leukocytes of 20 patients. The average EWBD in our study was estimated to be in the range of just 2.34–14.67 mSV and was significantly higher for linac than for orthovoltage RT. Based on our calculations, this variation of the EWBD between the radiation techniques was determined by differences in the SSD and half-value thickness. For otherwise identical parameterization, a higher SSD for linac RT caused an average 1.9-fold reduction of the integral dose compared to orthovoltage RT and, conversely, the higher half-value thickness for linac RT resulted in a six-fold increment of the integral dose than for orthovoltage RT. The impact of these two parameters resulted in a general significant 4.4-fold increase of the integral dose for linac RT compared to orthovoltage RT. This value also applied for calculated EWBD, since there were no significant differences in the distribution of the patients’ bodyweight between the two radiation modalities. We observed a general slight but significant increase of γH2AX foci per cell after RT for all patients but no difference between the RT techniques nor a correlation with the EWBD. 30 min after RT the numbers of excess γH2AX foci per cell were in a low range of 0 to 0.685, with a median of 0.104.

In our previous studies on the quantification of γH2AX foci in peripheral leukocytes of breast and prostate cancer patients after RT, we have shown linear dose–response relationships and good approximations of the administered whole-body dose based on ex vivo calibration data [26, 27]. However, cancer patients were exposed to significantly higher EWBDs compared to patients with benign diseases of the present study (Fig. 3d). According to our reference data on linear dose–response relationships of γH2AX foci in leukocytes at various times post exposure [26], the average frequency of 0.149 RI foci per cell after RT of all patients in the present work equates to a mean absorbed X‑ray dose of 15.1 mGy, which exceeds our calculated average EWBD of 6.77 mSv. But assuming that the number of foci of DSB repair proteins in peripheral leukocytes after RT is a quantitative measure of the patient’s dose burden and correlates with the risk of adverse side and late effects of medical radiation exposure, it is expectedly and significantly far lower in heel spur patients than for tumor patients. However, as we reported in our previous work [26, 27], the induction of DSBs in peripheral leukocytes during RT depends on various radiation and physiological parameters, which strongly limits such direct comparisons. Although the yield of γH2AX foci in peripheral leukocytes during RT of cancer patients is primarily governed by general RT parameters such as the planning target volume or the administered EWBD, we described volume- and dose-independent variations of radiation biomarkers in leukocytes among different RT techniques for breast cancer treatment which were heavily dominated by the absolute beam-on time [27]. Therefore, the radiation parameters between the two RT techniques of the present study, such as the field size or the dose rate, were adjusted as well as possible to achieve comparable exposure scenarios and beam-on times, to detect the impact of diverse out-of-field doses only. Besides, a strong dependency of foci induction in systemic leukocytes on physical variables such as the regional blood volume and kinetics of leukocyte circulation in the exposed anatomic region has to be considered for any comparative tactic [22, 24]. These confounding factors also greatly deteriorate the accuracy of radiation biomarkers for dose estimates after RT, in particular in the range of very low doses.

Taken together, using a biodosimetric approach to monitor the radiation burden of heel spur patients after the first fraction of RT with a single dose of 0.5 Gy administered with a 140-kV orthovoltage device or a 6-MV linac, we observed a marginal but significant overall increase in the DSB surrogate marker γH2AX in peripheral leukocytes, with no difference between the RT techniques. Both treatment modalities were associated with very modest radiation exposures and showed high and comparable analgesic effectiveness. Our data confirm the use of low-dose RT as an attractive treatment option for benign diseases.

## References

[1] Leer JW, van Houtte P, Davelaar J (1998). Indications and treatment schedules for irradiation of benign diseases: a survey. Radiother Oncol.

[2] Frey B, Hehlgans S, Rodel F, Gaipl US (2015). Modulation of inflammation by low and high doses of ionizing radiation: implications for benign and malign diseases. Cancer Lett.

[3] Heyd R, Tselis N, Ackermann H, Roddiger SJ, Zamboglou N (2007). Radiation therapy for painful heel spurs: results of a prospective randomized study. Strahlenther Onkol.

[4] Ott OJ, Jeremias C, Gaipl US, Frey B, Schmidt M, Fietkau R (2014). Radiotherapy for benign calcaneodynia: long-term results of the Erlangen dose optimization (EDO) trial. Strahlenther Onkol.

[5] Niewald M, Holtmann H, Prokein B, Hautmann MG, Rosler HP, Graeber S, Dzierma Y, Ruebe C, Fleckenstein J (2015). Randomized multicenter follow-up trial on the effect of radiotherapy on painful heel spur (plantar fasciitis) comparing two fractionation schedules with uniform total dose: first results after three months’ follow-up. Radiat Oncol.

[6] Ott OJ, Niewald M, Weitmann HD, Jacob I, Adamietz IA, Schaefer U, Keilholz L, Heyd R, Muecke R (2015). DEGRO guidelines for the radiotherapy of non-malignant disorders. Part II: painful degenerative skeletal disorders. Strahlenther Onkol.

[7] Surenkok S, Dirican B, Beyzadeoglu M, Oysul K (2006). Heel spur radiotherapy and radiation carcinogenesis risk estimation. Radiat Med.

[8] Leer JW, van Houtte P, Seegenschmiedt H (2007). Radiotherapy of non-malignant disorders: where do we stand?. Radiother Oncol.

[9] Damber L, Larsson LG, Johansson L, Norin T (1995). A cohort study with regard to the risk of haematological malignancies in patients treated with x-rays for benign lesions in the locomotor system. I. Epidemiological analyses. Acta Oncol.

[10] Jansen JT, Broerse JJ, Zoetelief J, Klein C, Seegenschmiedt HM (2005). Estimation of the carcinogenic risk of radiotherapy of benign diseases from shoulder to heel. Radiother Oncol.

[11] Pierce DA, Shimizu Y, Preston DL, Vaeth M, Mabuchi K (1996). Studies of the mortality of atomic bomb survivors. Report 12, Part I. Cancer: 1950–1990. Radiat Res.

[12] UNSCEAR (2000). Sources and effects of ionizing radiation. UNSCEAR 2000 report volume I: sources.

[13] Cole C, Seto C, Gazewood J (2005). Plantar fasciitis: evidence-based review of diagnosis and therapy. Am Fam Physician.

[14] Riepert T, Drechsler T, Urban R, Schild H, Mattern R (1995). The incidence, age dependence and sex distribution of the calcaneal spur. An analysis of its x-ray morphology in 1027 patients of the central European population. Rofo.

[15] Kriz J, Seegenschmiedt HM, Bartels A, Micke O, Muecke R, Schaefer U, Haverkamp U, Eich HT (2018). Updated strategies in the treatment of benign diseases—a patterns of care study of the german cooperative group on benign diseases. Adv Radiat Oncol.

[16] Muecke R, Micke O, Reichl B, Heyder R, Prott FJ, Seegenschmiedt MH, Glatzel M, Schneider O, Schafer U, Kundt G (2007). Demographic, clinical and treatment related predictors for event-free probability following low-dose radiotherapy for painful heel spurs—a retrospective multicenter study of 502 patients. Acta Oncol.

[17] Kase KR, Svensson GK, Wolbarst AB, Marks MA (1983). Measurements of dose from secondary radiation outside a treatment field. Int J Radiat Oncol Biol Phys.

[18] Dorr W, Herrmann T (2008). Second tumors after oncologic treatment. Strahlenther Onkol.

[19] Diallo I, Haddy N, Adjadj E, Samand A, Quiniou E, Chavaudra J, Alziar I, Perret N, Guerin S, Lefkopoulos D, de Vathaire F (2009). Frequency distribution of second solid cancer locations in relation to the irradiated volume among 115 patients treated for childhood cancer. Int J Radiat Oncol Biol Phys.

[20] Beels L, Bacher K, Smeets P, Verstraete K, Vral A, Thierens H (2012). Dose-length product of scanners correlates with DNA damage in patients undergoing contrast CT. Eur J Radiol.

[21] Depuydt J, Baert A, Vandersickel V, Thierens H, Vral A (2013). Relative biological effectiveness of mammography X-rays at the level of DNA and chromosomes in lymphocytes. Int J Radiat Biol.

[22] Lobrich M, Rief N, Kuhne M, Heckmann M, Fleckenstein J, Rube C, Uder M (2005). In vivo formation and repair of DNA double-strand breaks after computed tomography examinations. Proc Natl Acad Sci U S A.

[23] Rothkamm K, Balroop S, Shekhdar J, Fernie P, Goh V (2007). Leukocyte DNA damage after multi-detector row CT: a quantitative biomarker of low-level radiation exposure. Radiology.

[24] Sak A, Grehl S, Erichsen P, Engelhard M, Grannass A, Levegrun S, Pottgen C, Groneberg M, Stuschke M (2007). gamma-H2AX foci formation in peripheral blood lymphocytes of tumor patients after local radiotherapy to different sites of the body: dependence on the dose-distribution, irradiated site and time from start of treatment. Int J Radiat Biol.

[25] Schwab SA, Brand M, Schlude IK, Wuest W, Meier-Meitinger M, Distel L, Schulz-Wendtland R, Uder M, Kuefner MA (2013). X-ray induced formation of gamma-H2AX foci after full-field digital mammography and digital breast-tomosynthesis. PLoS One.

[26] Zahnreich S, Ebersberger A, Kaina B, Schmidberger H (2015). Biodosimetry based on gamma-H2AX quantification and cytogenetics after partial- and total-body irradiation during fractionated radiotherapy. Radiat Res.

[27] Zahnreich S, Ebersberger A, Karle H, Kaina B, Schmidberger H (2016). Quantification of radiation biomarkers in leukocytes of breast cancer patients treated with different modalities of 3D-CRT or IMRT. Radiat Res.

[28] Zwicker F, Swartman B, Roeder F, Sterzing F, Hauswald H, Thieke C, Weber KJ, Huber PE, Schubert K, Debus J, Herfarth K (2014). In vivo measurement of dose distribution in patients’ lymphocytes: helical tomotherapy versus step-and-shoot IMRT in prostate cancer. J Radiat Res.

[29] Zwicker F, Swartman B, Sterzing F, Major G, Weber KJ, Huber PE, Thieke C, Debus J, Herfarth K (2011). Biological in-vivo measurement of dose distribution in patients’ lymphocytes by gamma-H2AX immunofluorescence staining: 3D conformal- vs. step-and-shoot IMRT of the prostate gland. Radiat Oncol.

[30] Wang H, Adhikari S, Butler BE, Pandita TK, Mitra S, Hegde ML (2014). A perspective on chromosomal double strand break markers in mammalian cells. Jacobs J Radiat Oncol.

[31] Rowe CR, Sakellarides HT, Freeman PE (1963). Fractures of the os calcis a long-term follow-up study of 146 patients. JAMA.

[32] Johns HE (1961). Introduction to physics of radiobiology.

[33] Agarwal S, Tafel AA, Kanaar R (2006). DNA double-strand break repair and chromosome translocations. DNA Repair.

[34] Brand M, Sommer M, Achenbach S, Anders K, Lell M, Lobrich M, Uder M, Kuefner MA (2012). X-ray induced DNA double-strand breaks in coronary CT angiography: comparison of sequential, low-pitch helical and high-pitch helical data acquisition. Eur J Radiol.

[35] Kuefner MA, Grudzenski S, Hamann J, Achenbach S, Lell M, Anders K, Schwab SA, Haberle L, Lobrich M, Uder M (2010). Effect of CT scan protocols on x-ray-induced DNA double-strand breaks in blood lymphocytes of patients undergoing coronary CT angiography. Eur Radiol.

[36] Kuefner MA, Grudzenski S, Schwab SA, Wiederseiner M, Heckmann M, Bautz W, Lobrich M, Uder M (2009). DNA double-strand breaks and their repair in blood lymphocytes of patients undergoing angiographic procedures. Invest Radiol.

